# Cheaters divide and conquer

**DOI:** 10.7554/eLife.03371

**Published:** 2014-06-24

**Authors:** Kirsten Bomblies

**Affiliations:** 1 **Kirsten Bomblies** is in the Department of Organismic and Evolutionary Biology, Harvard University, Cambridge, United Stateskbomblies@oeb.harvard.edu

**Keywords:** speciation, meiotic drive, chromosomal rearrangements, recombination, *S. pombe*

## Abstract

Three ‘killer genes’ in one species of fission yeast act selfishly and keep it reproductively isolated from a closely related species.

**Related research article** Zanders SE, Eickbush MT, Yu J, Kang J-W, Fowler KR, Smith GR, Malik HS. 2014. Genome rearrangements and pervasive meiotic drive cause hybrid infertility in fission yeast. *eLife* **3**:e02630. doi: 10.7554/eLife.02630

**Image** Hybrid yeast (right) produce fewer viable spores and smaller colonies than either parent species (left, middle)

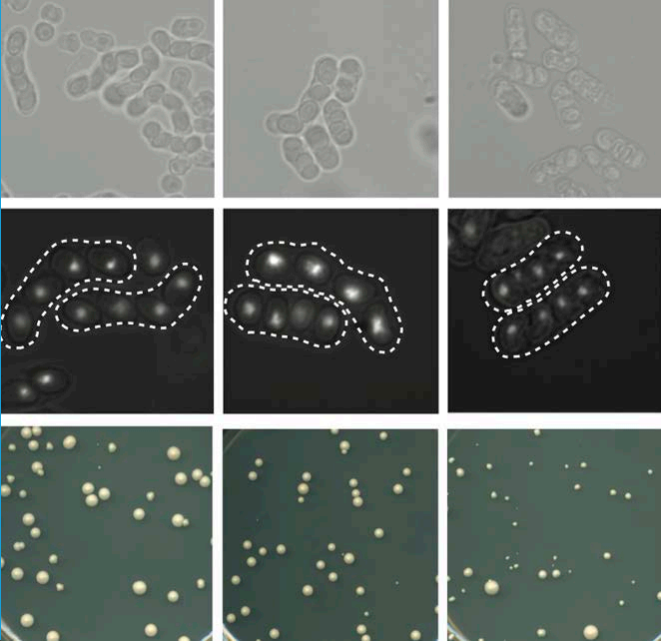

Cheaters who act selfishly to prosper at the expense of others are commonplace in the natural world, and genomes are no exception. Humans typically have two copies of each gene: we inherit one copy from our mother and the other from our father—and, if we have a child, we will pass on one of these copies essentially at random. However, there are genes or genetic elements that subvert the fairness of inheritance, often in creative and insidious ways, solely for their own benefit.

Some of these selfish genetic elements ensure that they get passed on to an individual's offspring more often than they should, by making their way into more than half of that individual’s gametes (e.g., sperm and egg cells in animals, or spores in fungi). Some of the best-studied examples of these genes are those that essentially commit a kind of fratricide to get ahead. Gamete killers directly cripple or kill any of their ‘sibling’ sperm or spores that did not inherit the killer gene, and these genes have been discovered in mice, flies and various fungi (Burt and Trivers, 2006).

Gamete killers are a subset of a broader class of 'meiotic drivers'. Meiotic drivers were originally defined as genes that could directly cheat when chromosomes are being segregated into the gametes (a process called meiosis). However, the term has now been more broadly applied to include any gene that subverts the fairness of inheritance by any means. Now, in *eLife*, Harmit Malik and colleagues at the Fred Hutchinson Cancer Research Center—including Sarah Zanders as first author—report that three meiotic drivers keep two yeast species reproductively isolated (Zanders et al., 2014).

By circumventing unbiased inheritance, meiotic drivers find shelter from being purged by natural selection acting on their hosts. Even meiotic drivers that cause a drop in fitness (in terms of survival or the number of offspring produced) can thus spread in populations. By eluding fitness-based selection, chromosomes carrying meiotic drivers can accumulate harmful mutations or structural rearrangements and this ‘baggage’ can be dragged along with the driver to higher frequency (Burt and Trivers, 2006). The negative effects of meiotic drivers select for other genes that suppress these effects, and this can initiate a molecular arms race between drivers and suppressors that is predicted to cause rapid evolutionary divergence of these genes. Because they can both rapidly diverge and compromise fertility, it has been suggested that meiotic drivers could cause related populations of organisms to become reproductively isolated (Hurst and Werren, 2001; McDermott and Noor, 2010)—something that might drive the generation of new species.

To date, evidence comes largely from studying the fruit fly *Drosophila*. But now, Zanders et al. have catalogued genetic elements that contribute to reproductive isolation between two closely related fission yeast species: *S. pombe* and *S. kambucha*. *S. pombe* has been studied as a model organism since the 1950s, while *S. kambucha* was isolated more recently from a fungus that has been used in China for centuries to make a drink called Che (Singh and Klar, 2002). These two species—which each have three chromosomes—can mate to produce hybrids, but these hybrids have very low fertility and often fail to produce viable spores.

Zanders et al. found that one region of the genome was the opposite way round (or inverted) in *S. pombe* (compared to *S. kambucha*), and that two essential genes had switched their positions in the genome of *S. kambucha*. Both of these rearrangements did affect fertility, but these differences were not sufficient to account for the extremely low spore production of hybrids of these two yeast species. After ruling out several alternatives, Zanders et al. discovered something remarkable: each chromosome in *S. kambucha* contains a spore killer gene. Spore killers are a type of gamete killer, and each encodes what is essentially a molecular poison. The spores that harbour a particular killer gene are immune to the respective poison, but the details of this immunity remain largely mysterious (though see Hammond et al., 2012). As such, a spore from the *S. pombe/S. kambucha* hybrid must receive all three killer genes by random segregation to be fully sheltered from all three poisons (Figure 1).

**Figure 1.**
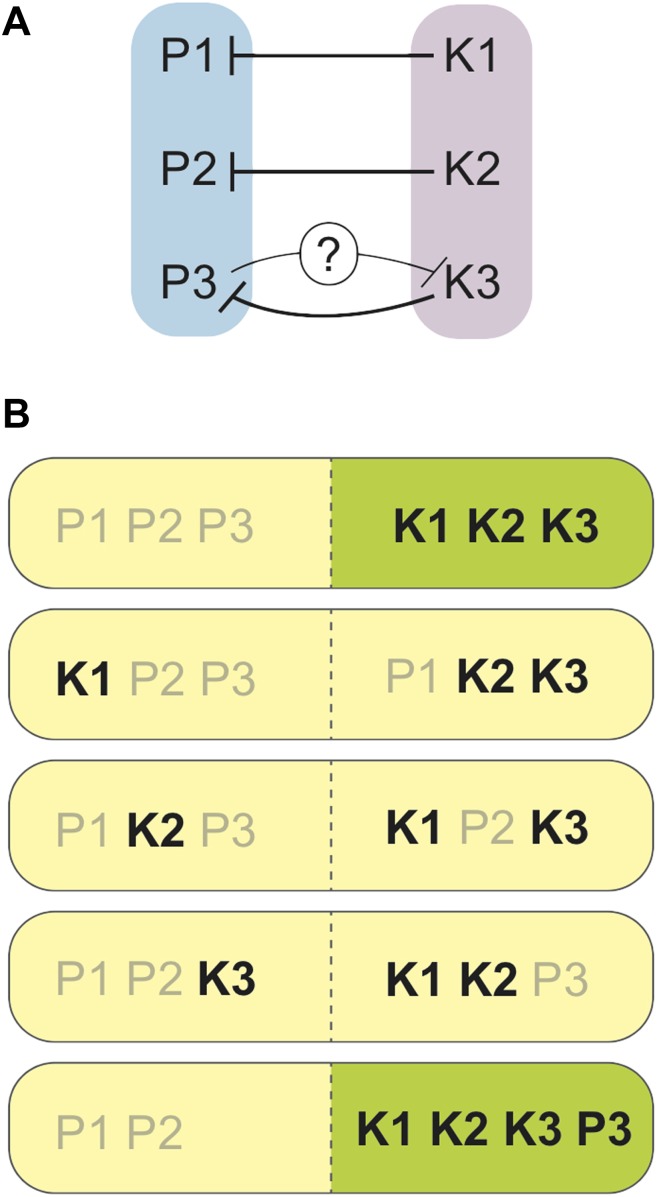
Spore killers interact and determine the survival of spores from *S. pombe/S. kambucha* hybrids. (**A**) Each chromosome in *S. kambucha* (K1-3) harbours a spore killer gene that works against (black lines) the corresponding chromosome in *S. pombe* (P1-3). Zanders et al. propose that the P3 chromosome in *S. pombe* contains a killer gene that works against (black line with question mark) the K3 chromosome in *S. kambucha*. (**B**) Combinations of chromosomes generated by random segregation during spore-production by the *S. pombe/S. kambucha* hybrid, including one aneuploid that inherits copies of chromosome 3 from both parents (bottom). Chromosome combinations predicted to yield viable spores are shown in green, and those predicted to die in yellow. Spore killers are labelled in black.

To add to the complication, two of the killer genes interact: the killer gene on chromosome 2 is stronger when there is also a killer gene on chromosome 3; however, the killer gene on chromosome 3 is weakened by that on chromosome 2. The mechanism of this interaction, and whether it directly results from the killer genes themselves, remains unknown. Furthermore, only hybrid spores that inherit versions of these two chromosomes from the same yeast species (either both from *S. pombe* or both from *S. kambucha*) are viable. This is because two essential genes have been swapped between chromosomes 2 and 3 in one of the parent species, and thus a spore must inherit these two chromosomes together, or die because it ends up lacking one or the other of these genes.

There is yet another twist: spores from the hybrids often carried both copies of chromosome 3, one originally from *S. pombe* and the other from *S. kambucha*. Having ruled out that hybrids might simply produce more aneuploids (spores with extra or missing chromosomes), Zanders et al. propose that there may be a weaker killer gene on the *S. pombe* version of chromosome 3, such that aneuploids carrying both versions of chromosome 3 are more likely to survive than spores with only the *S. kambucha* variant. Whether this is caused by a different version of the same gene, or by a distinct driver that arose independently on *S. pombe* chromosome 3, will be a very interesting follow-up question.

In summary, Zanders et al. provide an exciting milestone for research on meiotic drive systems and their potential links to speciation. The finding that multiple independent meiotic drivers can differ between even closely related species, can change the structure of genomes, and can also act together to cause a very strong fertility barrier, is an important insight. This study highlights that meiotic drivers need not be rare, and that they can both directly and indirectly affect multiple chromosomes. The identification of the underlying genes, and any suppressors that may exist, will not only allow us to understand the molecular mechanisms of spore killing, but may also clarify how meiotic drivers can arise repeatedly. This study reminds us that much remains to be learned about the dynamics of drivers and possible piggy-back effects on genome architecture, speciation, and extinction.
